# The Hospital Officer's Bookshelf

**Published:** 1913-01-18

**Authors:** 


### THE HOSPITAL OFFICER'S BOOKSHELF.

A Text-Book of Pathology. By J. G. Adami, M.A., M.D., F.R.S., and John McCrae, M.D., M.R.C.P. Illustrated with 304 engravings and 11 coloured plates. (London: Macmillan and Co., Ltd. 1912. Price 25s.)

The science of pathology as measured by the size of the treatises which claim to be fairly exhaustive has made great strides during the last decade. Whether the practical value to humanity at large of recent researches and laboriously constructed hypotheses is commensurate with this literary progress remains yet to be seen. But to the student of medicine it is indispensable that there should be available a one-volume text-book of reasonable proportions containing the foundations and the framework of this science displayed in plan and elevation and section with that clearness so necessary for the retention of a lasting and intelligible impression. In the text-book before us this object seems to have been fairly achieved by the writers. Being the authors also of a larger and exhaustive text-book in two volumes on the "Principles of Pathology," it would doubtless have been easy for them to have epitomised from this work and produced a more or less satisfactory digest. Fortunately they have avoided this danger. The result is a handbook, which, although somewhat expensive and weighty, should find favour with the medical student and the practitioner. It is by no means heavy reading, and has the merit of being remarkably succinct and intelligible. The first part of the book on general pathology is well done, and provides the student with an exceedingly serviceable and sufficiently comprehensive grounding in the facts and phenomena with which the science of medicine has been and is being built. It is obvious that much careful consideration has been necessary to keep within digestible limits the selection here to be found of the enormous mass of available material. The special and systematic pathology of the second portion of the book has, we gather, been written afresh almost independently of the matter in the authors' earlier and larger text-book. Here, again, the purpose of the book has been carefully kept in view—that is to say, the student and not the specialist is catered for. With regard to the illustrations, which are mostly original drawings from material in the McGill Medical Museum, these are distinctly helpful, being wonderfully clear and unobscured by excessive detail. This text-book—the latest in the field—is assured of a well-deserved popularity both in the Dominion and in this country.

A Manual of Immunity for Students and Practitioners. By Elizabeth T. Fraser, M.D. (Glasgow: James Maclehose and Sons. 1912. Pp. 199. Price 5s.)

The need for such a book as this cannot be gainsaid, and it is very pleasant to be able to place on record the efficient way in which Dr. Fraser has supplied this need. The general practitioner and the student will find in this modest volume exactly what they want to know of the subject of immunity. This subject is one which, unfortunately, has acquired a reputation for complexity in excess of its intrinsic difficulties of comprehension. This is largely due to the introduction of so many new terms which in themselves tend to discourage the busy practitioner from proceeding further, and thus so many doctors are forced, as the author says in the preface, to remain in the position of a passive transmitter of ideas which are entirely beyond his grasp. The remedy for this is a little patience and Dr. Fraser's book, wherein are set out in an easy and clear fashion the essential facts of the numerous reactions concerned directly and indirectly with the question of immunity. The reading is the reverse of dry, and the practical applications of the interesting feats performed by agglutinins, precipitins, vaccines, etc., are well described. The subject of the moment—namely, tuberculin—naturally receives attention, but practically everything bearing on immunity reactions is adequately but succinctly dealt with. This is a practical book and is not abstruse or exhaustive. Dr. Fraser presents a plain unvarnished tale in a pleasant style and has not forgotten either an index or a glossary.

Isotonic Sea-Water Injections: Practical Hints for Treatment. By Arthur Gregory Sandberg, M.D., and Dorothea Mary Tudor, M.B. (London: The Scientific Press, Limited. 1912. Price 2s. 6d.)

Those who are impressed with the value of the work done at the Quinton Polyclinic will be glad to know of the existence of this little book, which may perhaps help to confirm their faith. Those who still remain sceptics are here, as elsewhere, hardly likely to be converted. The range of cases described as being benefited or cured by this "plasma" is exceedingly wide, comprising all sorts of conditions from alopecia to lymphosarcoma of the tonsil. It is interesting to note that at this Polyclinic a swelling of the tonsil is discovered "under the laryngoscope." The efficacy of this method of "marine healing born from the theory of the descent of man from the ocean" appears to the authors proved beyond cavil, especially in cases of enteritis and marasmus. But more than this, it "has freed the doctrine of evolution from mechanism and infused it with a soul"; perhaps incidentally it has also directed attention to the efficacy of saline infusions not made from sea-water. Still there is nothing like hearing an enthusiastic minority present its case, and we heartily commend it to all who want to know what the protagonists of the Quinton Polyclinic have to say on their own behalf.

The Malthusian Limit. By Edward Isaacson. (London: Methuen and Co., Limited. 1912. Pp. 215. Price 3s. 6d.)

The author in this somewhat prolix volume deals exhaustingly with a theory of a possible static condition for the human race. Starting with the law of Malthus, that the natural increase of mankind will ultimately outstrip the available means of subsistence, the writer evolves a new social system in which there are to be two classes of human beings—the fecund class, from which the race is reproduced, and the surplus class, whose relations to society are those of individuals only. It would be impossible to do justice in a brief space to the way in which through over two hundred pages the ramifications of a formula like this are discussed. It is only fair to state that those who care for such speculative sociology will find in this book much that is interesting. No propaganda is intruded, and none knows better than the author that such a clear-cut scheme could not be put into practice for a dozen centuries or so. But it furnishes a new perspective, and may throw some light on practical questions. At any rate, those who read this book will not have wasted their time, whatever they may think of the ideas that meet them.

A Manual of Infectious Diseases Occurring in Schools. By H. G. Armstrong, M.R.C.S., and J. M. Fortescue-Brickdale, M.A., M.D. (Bristol: John Wright and Sons, Ltd. 1912. Price 3s.)

It is with real pleasure that we are able to express a favourable opinion upon this modest volume. A manual of this type, dealing with a class of diseases of such great importance in schools, cannot fail to serve a useful purpose. The Association of Preparatory Schools, under whose auspices this book has been issued, has fortunately secured the services of two medical men who are able to write on the subject of infectious diseases from their own personal experiences, and the consequence is that the manual is essentially practical and often more accurate in certain particulars than are the descriptions to be found in some of the text-books of medicine, where the information on these points is not infrequently second-hand. As this book is intended primarily for the instruction of masters and mistresses of schools, the descriptions of the various fevers are rightly straightforward and free from confusing abstruseness. Nevertheless, the subject-matter has been brought up to date, and certain ancient platitudes have been providentially omitted. Useful figures are given regarding the percentages of children who have already had the several specific fevers at the public school age, and a short chapter is included on the subject of school epidemiology. In the chapter on measles we consider that too much attention has been devoted to the variation in weight during the incubation period—the so-called Meunier's sign—and it is perhaps unnecessary to suggest, as is done on the plate depicting a child with measles, that this rash resembles that of chicken-pox or smallpox. Mr. R. W. Doyne has contributed a short practical chapter on the infectious diseases of the eye, and this, together with Dr. Aldersmith's excellent description of ringworm, adds greatly to the value of the manual. The book can be confidently recommended to schoolmasters, and indeed to all those working at school inspection, medical officers included.

